# Increasing awareness of gynaecological cancer symptoms: a GP perspective

**DOI:** 10.3399/bjgp14X680161

**Published:** 2014-05-27

**Authors:** Ruth EC Evans, Melanie Morris, Mandeep Sekhon, Marta Buszewicz, Fiona M Walter, Jo Waller, Alice E Simon

**Affiliations:** Research associate, Health Services Research, School of Health Sciences, City University, London.; Research fellow in epidemiology, Health Behaviour Research Centre, Department of Epidemiology and Public Health;; Health Services Research, School of Health Sciences, City University, London.; Research Department of Primary Care and Population Health, University College London, London.; Primary Care Unit, Department of Public Health and Primary Care, University of Cambridge, Cambridge.; Health Behaviour Research Centre, Department of Epidemiology and Public Health;; Health Services Research, School of Health Sciences, City University, London.

**Keywords:** early detection of cancer, general practice, patient education, primary health care

## Abstract

**Background:**

In the UK there has been an effort, through the National Awareness and Early Diagnosis Initiative (NAEDI), to increase early stage diagnoses and ultimately cancer survival. Encouraging early symptom presentation through awareness-raising activities in primary care is one method to achieve this goal. Understanding GPs’ views about this type of activity, however, is crucial prior to implementation.

**Aim:**

To describe GPs’ attitudes to raising public awareness of gynaecological cancers, and their views about the potential impact on primary care services.

**Design and setting:**

An online survey with a convenience sample recruited from 1860 UK general practices.

**Method:**

An invitation was emailed to GPs via practice managers and included a weblink to a draft education leaflet and an online survey about the impact of sending a leaflet giving information about symptoms associated with gynaecological cancers to all women on GPs’ lists. Participants could offer additional free text comments which were coded using content analysis.

**Results:**

A total of 621 GPs participated. Most (77%, 477) felt that raising awareness of cancers was important. Only half (50%, 308), however, indicated that they would distribute such a leaflet from their practice. Barriers to implementation included concerns about financial costs; emotional impact on patients; increased demand for appointments and diagnostic services, such as ultrasound.

**Conclusions:**

GPs were generally positive about an intervention to improve patients’ awareness of gynaecological cancers, but had concerns about increasing rates of presentation. There is a need for research quantifying the benefits of earlier diagnosis against resource costs such as increased consultations, investigations, and referrals.

## INTRODUCTION

There is increasing evidence that cancer survival can sometimes be improved by earlier diagnosis, which is potentially achievable through symptomatic individuals presenting earlier to health professionals.^1^ This is true for a number of cancers including most of the gynaecological cancers, which have a combined annual incidence second only to breast cancer in the UK (*n* = 19 631, in 2010).^2^–^5^ Thomson and Forman^6^ concluded that earlier diagnosis of cervical and uterine cancers could reduce the survival gap between England and European averages, and differences in ovarian cancer survival could also be reduced by earlier diagnosis, although care factors after diagnosis also play a role.

Encouraging earlier presentation of cancer symptoms could be achieved in a variety of ways, including education and information provision (for example, multimedia campaigns) as well as changes in healthcare provision (for example, access to appointments). In the UK there has been a coordinated effort between the Department of Health and cancer charities to develop programmes of cancer awareness-raising activities through the National Awareness and Early Diagnosis Initiative (NAEDI). Increasing public cancer awareness via information provision is a key strategy for increasing the rates of earlier presentation.^7^ The assumption is that education leads to symptom awareness, which is a necessary prerequisite to help-seeking behaviour. There is some evidence from evaluation data assessing the impact of UK multimedia cancer awareness campaigns that supports this assumption.^8^,^9^ Changes in behaviour, such as increased presentations to the GP^8^,^9^ and attendance at cancer screening,^10^ have been recorded and associated with an increase in urgent referrals for investigations.^8^ Evidence that more cancers are being detected, and at an earlier stage, is preliminary. In one encouraging example, a regional evaluation of a lung cancer awareness campaign found a significant increase in the number of small cell cancers staged as ‘limited’ (that is, confined to one lung and relatively early in presentation).^8^

Some clinicians have been critical of efforts to educate the public about cancer symptoms as a means of promoting early detection. The impact of education has been labelled by some as ‘the curse of awareness’,^11^ with concerns voiced that campaigns may lead patients to have an exaggerated perception of personal risk and increased anxiety about their health. This may lead to the ‘worried well’ flooding general practice, resulting in unnecessary investigations to reassure patients that they do not have cancer.^12^ There have been no systematic attempts to quantify the extent of GPs’ concerns, although GPs are recognised as influential in raising the profile of cancer symptoms among their patients.^7^ As gatekeepers to secondary care and diagnostic services, GPs are key stakeholders in developing educational initiatives designed to increase help-seeking behaviour. GPs’ concerns regarding these initiatives, therefore, must be identified and dealt with.

**How this fits in**

Raising cancer awareness among the public is a recent UK government strategy to encourage symptomatic patients to present promptly in primary care. This study highlights the role of GPs as key stakeholders in individualised interventions located within primary care, and reports the findings of a GP consultation at the development stage of a leaflet intervention aimed at increasing awareness of gynaecological cancers. The focus of this study was to identify and quantify levels of support and barriers to the implementation of such an intervention among GPs.

In this study, a leaflet was developed to encourage the earlier presentation of women with gynaecological cancer symptoms. The leaflet, to be directly mailed to women aged 40 years on GPs’ lists contained information about gynaecological cancers including symptoms and signs, a symptom checklist for patients to complete and take to a GP appointment, and acknowledged common help-seeking barriers and suggestions of how to overcome these. In addition, one panel of the leaflet included a ‘letter’ from the GP inviting women to make an appointment if they had any of the symptoms highlighted in the leaflet. Leaflets are a commonly used and relatively inexpensive, but pragmatic approach to imparting health information. They can be used as a stand-alone intervention or embedded within a more extensive multimedia social marketing campaign.^13^

To assess GPs’ attitudes to raising public awareness of gynaecological cancers and their views about the impact on primary care services, a sample of GPs were invited to comment on a draft version of the leaflet and to give their views about the likely effect of distributing it.

## METHOD

This web-based survey was delivered to a convenience sample of GPs. The design was categorised as service evaluation.

In October 2011 an email was sent to 1860 practice managers in the UK via the following organisations: the Primary Care Research Network (PCRN), the General Practice Research Framework (GPRF), and the Society for Academic Primary Care (SAPC). The 1860 practices represented approximately 20% of registered general practices within the UK (excluding Northern Ireland).^14^

Practice managers were asked to forward the email to all doctors (including locums and trainees) working in their surgery. The email gave details of the study, a weblink to the leaflet and to a short (approximately 15-minute) questionnaire. A small incentive was offered, with a prize draw giving the opportunity to win £75 in vouchers or six bottles of sparkling wine being held 3 months after the initial email.

Participation was anonymous, and informed consent was assumed if responders proceeded to the questionnaire. Two reminders to participate were sent to practice managers at 3–4-week intervals. The website was live between November 2011 and January 2012.

### Measures

The survey was developed in collaboration with the GP researchers in this study, before being piloted with two further independent GPs. There was a mixture of closed and open-response (free text) questions. The questionnaire and items included in the analysis are available from the authors on request. The questions included were broadly categorised into three themes.

#### Exploring perceived need for, and commitment to, raising awareness of gynaecological cancer symptoms

Participants were asked what they thought could prevent women from presenting early with symptoms of gynaecological cancers and what suggestions they had for overcoming these barriers. Commitment to raising awareness was assessed using responses to two statements using a 5-point agreement scale: ‘Personally I think that raising awareness of the symptoms of gynaecological cancers is a high priority’, and ‘I expect that sending out this leaflet to women in my practice would be considered a priority by the practice team’. The 5-point scale was later categorised into three: ‘strongly disagree/disagree’, ‘unsure’, and ‘agree/strongly agree’. Responders were also asked whether they had systems in place to encourage women with possible gynaecological cancer symptoms to present at their practice with response options of: ‘yes’, ‘no’, or ‘don’t know’.

#### Support for the leaflet and barriers to implementation

Responders were asked to indicate whether they would agree, in principle, to send the leaflet from their practice (‘yes’, ‘no’, and ‘maybe’), and about the possible impact of doing this by rating their agreement with nine statements (on the same agreement scale as above, later collapsed into three), for example ‘I expect that sending out this leaflet to women in my practice would be time consuming’.

Responders were asked about the local availability of diagnostic services for gynaecological cancers and to indicate whether diagnostic testing was available in-house (‘yes’ or ‘no’). Finally, responders rated ease of access for each of these investigations: CA125 serum test, abdominal ultrasound, transvaginal ultrasound, and colposcopy.

#### Practice and GP characteristics

Participants provided demographic details about themselves and their practice including: responder age, sex, and ethnicity, practice list size, location (region within the UK as well as practice setting: whether it was ‘urban’, ‘suburban’, ‘rural’, or ‘none of these’), ethnic diversity of the patient list, and whether there was a gynaecological specialist at the practice. For the analyses, ethnic diversity of the patient list was collapsed from four categories (‘very mixed across ethnicities’, ‘a mix of two main ethnicities’, ‘a majority of one ethnicity’, and ‘almost exclusively one ethnicity’) into two categories: ‘mixed ethnicities’ and ‘majority of one’; and GP ethnicity was collapsed into two categories: ‘white’ and ‘not white’.

### Analysis

Content analysis^15^ was used to convert free text comments into numerical data that could be summarised alongside the quantitative responses. Data were analysed using IBM SPSS (version 20). Proportions and frequencies were calculated and group differences were tested using χ^2^ analyses and ANOVA. Quotes from the free text comments illustrate findings described in the results.

For the content analysis, a coding framework was developed to describe free text responses in terms of the range of beliefs expressed, and the numerical frequency with which they occurred. A belief was defined as a specific idea, and could be a single word or several sentences of text coded for manifest meaning. Beliefs for each question were coded separately and recorded only once per participant per question. The framework was developed by two researchers using a thematic approach.^16^ A cyclical process of coder training, testing, and revision was followed to refine the categories and codes. Inter-rater reliability was calculated (IRR; Cohen’s κ) based on data from 10% of the sample (*n* = 60). All the κ values were within the acceptable range (all >0.8, *P*<0.001 with the exception of one, ‘suggestions for overcoming barriers’ which was 0.7, *P*<0.001).^17^ The remaining data were then coded using NVivo 9. After coding it was possible to produce numerical tables where columns represented beliefs and rows represented participants. Each cell contained either a 0 or 1 to represent absence or presence of a belief.

## RESULTS

A total of 621 GPs responded. The response rate could not be calculated as the numbers of GPs recruited and working at each practice could not be identified. Most responders worked in England (99%, Table 1). Comparison with statistics for England^18^ suggests the sample over-represented the 40–49 years age range (37% versus 32% nationally), and that there was a greater proportion of female GPs in the sample than would be expected (58% versus 46%), although retainers and registrars were not included in the national figures. The mean list size in the sample (10 039) was bigger than the national average of 6651. This may reflect the larger representation of practices from the Midlands and Eastern England where there are typically larger practice sizes.

**Table 1.** Demographics of participants and their practices^a^

**GP characteristics (*n* = 621)**	**% (*n*)**
**Age, years**	
<30	1.6 (10)
30–39	19.8 (123)
40–49	37.4 (232)
50–59	35.3 (219)
60–69	4.2(26)
≥70	0.2 (1)

**Sex**	
Male	41.1 (255)
Female	58.3 (362)

**Ethnicity**	
White	85.7 (532)
Black/African/Caribbean/black British	1.1 (7)
Asian/Asian British	9.3 (58)
Mixed/other	2.3 (14)

**Practice characteristics (*n* = 621, some participants may have come from same practice)**	

**List size mean**	10 039.86^b^ (SD 7812.1)
Smaller than national average 6651	29.8 (185)
Larger than national average 6652	68.6 (426)

**Location of GP practice**	
East England	16.4 (102)
London and South East	18.9 (117)
Midlands	22.8 (141)
North East	3.4 (21)
North West	2.7 (17)
South West	16.6 (103)
York and Humber	15.3 (95)
Scotland	0.8 (5)
Wales	0.2 (1)

**Practice setting**	
Urban	36.6 (227)
Suburban	36.6 (227)
Rural	20.6 (128)
None of these	5.8 (36)

**Ethnicity diversity of patient list**	
Mixed	21.4 (133)
A majority of one	78.1 (485)

**Gynaecological/women’s health specialist in practice**	
Yes	50.1 (311)
No or don’t know	49.4 (307)

a Numbers may not add up to 621 because of missing data;

b *Median for the sample is* n *= 9000.*

### Exploring the need and support for raising awareness of gynaecological cancer symptoms

#### Reasons for longer time to presentation

Most GPs (86%, 532/621) offered at least one reason for late presentation among women with gynaecological cancer symptoms (Table 2). Only a few (2%, 14/621) reported that women attended promptly with such symptoms. The most often cited cause of longer time to presentation was low awareness (43%, 267/621) resulting in patients failing to understand the significance of symptoms. The vague nature of many of the symptoms associated with gynaecological cancers, particularly ovarian cancer, was highlighted as a key cause of longer time to presentation (20%, 124/621), for example:

‘[symptoms] ... *are very non-specific and confused with events such as menopause, gaining weight or* [confused with] *GI symptoms.’*

(#433)

**Table 2.** Reasons offered by GPs, in free text, for late presentation of potential gynaecological cancer symptoms listed in rank order (*n* = 621)

**Barrier**	**% sample (*n*)**
Gynaecological cancer awareness	43.0 (267)
Embarrassment	32.0 (199)
How cancer symptoms present	20.0 (124)
Fear/anxiety/stigma of cancer	18.7 (116)
Reluctance to engage with health professional/health not prioritised	12.7 (79)
Access to health care	11.4 (71)
Socioeconomic factors (for example, education/culture)	5.2 (32)
Reluctance to have an examination	4.7 (29)
Denial	3.7 (23)
False reassurance from a clear smear result	1.9 (12)
Perceive self to be low risk	1.1 (7)
Wait and see approach to symptoms	0.8 (5)
Fatalism	0.5 (3)
Acceptance of pain	0.3 (2)
Participants who did not offer an answer	9.8 (61)

Many participants cited more than one barrier so percentages do not add up to 100.

Another often cited barrier was patients’ embarrassment (32%, 199/621), including embarrassment about the urogenital area, symptoms potentially related to sex (for example, postcoital bleeding), and the possibility of discussing symptoms with a male GP. Other emotional barriers identified by GPs included fear, anxiety, and stigma associated with a cancer diagnosis (19%, 116/621). These barriers were thought to lead to denial and ultimately a longer time to presentation, for example:

‘*They may fear cancer so bury their head in the sand and avoid the GP.’*

(#507)

#### Suggestions for improving early presentation

Most GPs (70%, 433/621) offered ideas about how to improve early presentation. The most common suggestion was patient education (51%, 316/621), through national media and local community campaigns, practice specific initiatives, or opportunistic conversations with patients. Other suggested solutions included improved healthcare provision (14%, 90/621), such as increasing access to appointments and to female doctors, as well as encouraging all staff to be more approachable, for example:

‘*Good general practice and approachable primary care (includes GPs, nurses, receptionists etc).’*

(#213)

Health professional training (3%, 16/621) was suggested by a few as a way to:

*‘Ensure all GPs are aware of ALL symptoms and keep up-to-date with protocols and pathways.*’

(#280)

Overall, 106 GPs (17%) specifically cited the educational leaflet as a useful intervention:

*‘I think leaflets such as this can convince them* [women] *of the importance of presenting.’*

(#199)

One-quarter of responders (25%, 152/621) reported that they already had systems in place to encourage women with possible gynaecological cancer symptoms to present promptly at their practice, for example:

*‘Posters in waiting room. Give health promotion and advice at contraceptive and smear appointments.*’

(#722)

#### Levels of commitment to raising awareness and support for using the gynaecological cancers information leaflet

Most GPs (77%, 477/621) believed that raising awareness of gynaecological cancers was a priority in general terms. For their own practice, however, most were more cautious and only 16% (100/621) agreed that raising awareness would be a priority for them.

Half the sample (50%, 308/621) agreed, in principle, that they would send out the research leaflet from their practice.

A minority (12%, 77/621) said they would not use the leaflet, and around one-third (37%, 229/621) were undecided (‘maybe’). Endorsement was not related to GP characteristics of sex (χ^2^ (2 degrees of freedom [df]) = 2.96, *P* = 0.228), age (*F* (2 df) = 2.697, *P* = 0.068), or ethnicity (χ^2^ (2df) = 0.274, *P* = 0.874), nor to practice characteristics of practice setting (χ^2^ (4 df) = 2.94, *P* = 0.568), ethnic diversity of patients (χ^2^ (2 df) = 1.092, *P* = 0.579), or practice size (χ^2^ (2 df) = 1.611, *P* = 0.447). GPs who reported having a gynaecological specialist in the practice, however, were more likely to agree to send out the leaflet than those from practices with no gynaecological specialist (55% versus 45%; χ^2^ (2 df) = 6.133, *P* = 0.047).

As shown in Table 3, a greater proportion of GPs who said they would send out the leaflet thought that it would impact positively on women.

**Table 3.** Overall perceived impact of the leaflet on patients and grouped by GPs’ inclination to use the leaflet (*n* = 621)

	**GPs’ inclination to use the leaflet % (*n*)**			**Group differences between proportions agreeing**	
	
	**Yes**	**Maybe**	**No**	**χ^2^ (df)**	***P*-value**
**Would inform women about how to deal with symptoms they may have**					
Agree	84.0 (258)	62.9 (144)	43.4 (33)	68.8 (4)	<0.0001
Unsure	9.8 (30)	19.7 (45)	21.1 (16)		
Disagree	6.2 (19)	17.5 (40)	35.5 (27)		

**Has an appropriate level of encouragement**					
Agree	83.4 (256)	53.8 (121)	26.3 (20)	142.4 (4)	<0.0001
Unsure	12.1(37)	28.9 (65)	23.7 (18)		
Disagree	4.6 (14)	17.3 (39)	50.0 (38)		

**Would help patients talk about a difficult subject**					
Agree	91.9 (283)	69.0 (158)	52.6 (40)	90.9 (4)	<0.0001
Unsure	7.1 (22)	26.6 (61)	30.3 (23)		
Disagree	1.0 (3)	4.4 (10)	17.1 (13)		

**Would increase women’s fears of gynaecological cancer**					
Agree	53.7 (165)	76.0 (174)	78.9 (60)		
Unsure	28.0 (86)	14.8 (34)	14.5 (11)	36.1 (4)	<0.0001
Disagree	18.2 (56)	9.2 (21)	6.6 (5)		

df = degrees of freedom.

### Barriers to using the gynaecological cancers information leaflet

One-third (36%, 224/621) of responders gave one or more free text comments in response to questions about whether they were prepared to distribute the leaflet, and their perceived barriers to its implementation are grouped by theme below. There were 637 comments in total. A few (7%, 42/637) were positive endorsements or support for this intervention, whereas about half (46%, 295/637) described the perceived potential negative impact of the leaflet on patients or practices. The remainder referred specifically to the content (13%, 84/637) and suggestions for change to either the leaflet or how it was disseminated (22%, 138/637).

A few GPs felt that they needed additional information (4%, 25/621) before making a decision about the leaflet. Although for some this was about seeking the opinions of colleagues, for others it was about needing to know the likely impact, for example:

‘I would want to see some evidence that there is a benefit, preferably from a good quality RCT showing that the leaflet actually results in a benefit (gynaecological cancers diagnosed sooner with corresponding clinical benefit) greater than the harms (costs, unnecessary investigations and consequences of that, stress to women).’

(#213)

#### Financial concerns

As reported in Table 4, over half the GPs agreed that it would be easy to mail out the leaflet (56%, 350/621), but most thought it would be costly (72%, 445/621).

**Table 4.** Perceived impact of the leaflet on GP practices with a total score and grouped by GP’s inclination to use the leaflet (*n* = 621)

**Sending the leaflet would be ...**	**GPs’ inclination to use the leaflet % (*n*)**			**Group differences between proportions agreeing**	
	
	**Yes**	**Maybe**	**No**	**χ^2^ (df)**	***P*-value**
**... easy**					
Agree	70.1 (213)	46.9 (107)	36.8 (28)	65.5 (4)	<0.0001
Unsure	15.8 (48)	23.7 (54)	10.5 (8)		
Disagree	14.1 (43)	29.4 (67)	52.6 (40)		

**... time consuming**					
Agree	70.0 (212)	79.9 (183)	87.0 (67)	13.7 (4)	0.0080
Unsure	10.9 (33)	8.3 (19)	6.5 (5)		
Disagree	19.1 (58)	11.8 (27)	6.5 (5)		

**... onerous on staff**					
Agree	56.0 (169)	73.4 (168)	77.6 (59)	23.4 (4)	<0.0001
Unsure	20.5 (62)	13.1 (30)	10.5 (8)		
Disagree	23.5 (71)	13.5 (31)	11.8 (9)		

**... unlikely to lead to many additional appointments**					
Agree	8.5 (26)	11.4 (26)	15.6 (12)	11.5 (4)	0.0220
Unsure	20.3 (62)	11.4 (26)	11.7 (9)		
Disagree	71.1 (217)	77.3 (117)	72.7 (56)		

**... costly**					
Agree	68.6 (208)	78.4 (178)	76.3 (58)	7.0 (4)	0.1350
Unsure	19.8 (60)	14.5 (33)	14.5 (11)		
Disagree	11.6 (35)	7.0 (16)	9.2 (7)		

df = degrees of freedom.

Alternatives to a mail out were suggested and these included: having the leaflets available in the practice to pick up, displaying the information as a poster, sending the leaflet with other correspondence, distributing leaflets electronically, or posting the leaflet on the practice website.

#### Impact on patient behaviour

About two-thirds of the GPs (65%, 404/621) believed that the leaflet could increase patients’ fear of gynaecological cancers, and three-quarters (450/621) felt that it would lead to a significant increase in the number of appointments made (Tables 3 and 4), at a time when resources were already tight and there was pressure to reduce budgets. One responder noted that:

‘*None of us are looking for extra work and this represents one of many invitations from different interest groups asking people to make appointments which are already difficult enough to obtain’*

(#303)

#### Impact on diagnostic services

Most (85%, 527/621) said that they felt that their current access to diagnostic services locally was ‘just about right’ or ‘more than enough’ to meet demand. Similarly, most felt that access to specific investigations was ‘extremely’ or ‘quite’ easy. Just under half of the responders reported that they could carry out diagnostic testing within their practice (43%, 267/621). Free text comments revealed that some GPs (9%, 53/621) were concerned about waiting times, particularly for non-urgent ultrasounds, where clinical presentation does not warrant referral via the 2-week wait rule:

‘*Ease of access: we just have to write a request and wait. Speed of access: that’s a different and rather more pertinent question.*’

(#218)

Only a few GPs (3%, 17/621) felt that secondary services would be unable to cope with any increase in demand, leaving patients alerted to the possibility of a problem but having to wait significant lengths of time before accessing diagnostic services. As a result, some felt that resources should be directed to diagnostic services rather than raising awareness:

‘I think it is almost more important to provide the imaging services so that when GPs are concerned they can send the patients for hopefully a reassuring ultrasound within a week or two.’

(#211)

## DISCUSSION

### Summary

Despite general support for raising awareness of cancers and educational leaflets, only a small proportion of the GPs considered this activity a priority for their practice. Reservations about awareness campaigns, and sending leaflets out in particular, included concerns about raising patient anxiety, the possible pressure on appointments and referrals, and the financial impact. Some reservations stemmed from the need for any intervention to be evidence-based and there is, as yet, only limited published evidence that links raising patient awareness with earlier stage at diagnosis and no evidence as yet of any impact on mortality rates.^8^ GPs are best placed to give informed views on how to design and implement interventions that target their practice populations effectively.

### Strengths and limitations

This is the first study that gives a voice to an important stakeholder group in the debate about the benefits and costs of implementing individualised cancer awareness education within primary care.

Approaching GPs directly may be more effective in achieving a more representative sample than achieved here: this sample comprised mostly female responders, which is perhaps not surprising in a study about cancers affecting women. This may have led to an overestimation of the priority attached to raising awareness of gynaecological cancers, and it could explain the discrepancy between the numbers who perceived it to be a personal rather than a practice priority.

Health professional surveys typically have a low response rate,^19^,^20^ but the number of participants in this survey compared with the potential numbers contacted was possibly lower than similar studies.^21^ Despite this, and it not being possible to calculate an accurate response rate, the decision to contact GPs via professional organisations was beneficial in rapidly gaining access to a large number of GPs across the UK.

### Comparison with existing literature

The reasons offered by GPs for why women with gynaecological cancer symptoms may present late in primary care are broadly similar to those previously discussed in the literature.^22^–^24^ Patient education was the most frequent suggestion for improving early presentation. GPs were understandably concerned, however, about the potential for a negative impact on patients. Concerns about raising patient anxiety as a result of education are not supported by evidence in the literature, which shows little or no impact on ‘current state’ anxiety in samples exposed to cancer awareness information.^25^–^27^ GP education in relation to cancer awareness should include this information. GPs also expressed concern that increasing symptom awareness could lead to unnecessary investigations and associated patient distress. Evidence from existing gynaecological cancer literature comes from trials of population screening for ovarian cancer and suggests that when women receive an abnormal result which on subsequent testing is normalised (false positive) they do not report long-term distress.^28^ There is the possibility, however, that these false-positive results lead to unnecessary surgical intervention which has a risk of complications.^29^ Promoting symptom awareness as reported here is not the same as population screening; GPs triage patients deciding who has further investigations, and so, theoretically, false-positive results and their consequences should be fewer, but this needs to be quantified through research.

In terms of cost, the increase in cost of more frequent investigations must be offset against any potential reduction in cancer morbidity and mortality. The potential economic impact of earlier diagnosis initiatives has been modelled for some cancers (including breast, colorectal, lung, and melanoma), leading to the conclusion that such initiatives would at least be cost-effective, although not cost-saving.^30^ The intention of awareness-raising activities is to increase the number of symptomatic presentations, but GPs understandably expressed concern that such interventions could generate a large number of unnecessary presentations. Research that quantifies the likely increase in appointments and their outcome in terms of positive diagnoses is still needed. Preliminary work from national campaigns using the ‘Be Clear on Lung Cancer’ branding, suggests that the increase in patient consultations is manageable (fewer than three additional presentations per practice per week).^31^ However, the individual-level approach defined in this study, with a targeted leaflet sent to patients on GPs’ lists, has not yet been assessed for its impact on patient behaviour. Previous studies using this approach have only looked at intermediate patient outcomes; that is, those that precede behaviour, such as changes in beliefs or knowledge.^32^ Potentially, this type of GP-endorsed direct mailing may produce a more powerful behavioural response. This hypothesis is now being tested by distributing the gynaecological cancer leaflet described in this study to 10 pilot practices in and around North London.

Cancer symptoms can be non-specific and diagnostic delays have been linked to misattributions by clinicians and a failure to investigate symptoms that appear ambiguous.^22^,^33^,^34^ An increase in cancer-specific training and widespread implementation of decision support tools for GPs may increase their confidence in identifying patients with potential cancer symptoms. Significant progress has been made in this area recently,^35^,^36^ particularly in relation to the risk assessment tools (RATs) developed by Hamilton *et al.*^37^ (lung and bowel cancers), and Hippisley-Cox and Coupland’s ‘QCancer’ risk scores^38^,^39^ (which included gynaecological cancers in women), although how well they work in practices in general is not yet established. An effective approach could be to run public awareness campaigns in conjunction with GP education programmes. This approach was recently used to increase presentations to GPs and referrals for chest X-rays, and led to a 27% increase in lung cancer diagnoses in intervention areas compared with controls, although this difference was not statistically significant.^9^

One important issue included in the current survey was access to diagnostic tests. Improving direct GP access to diagnostic tests for cancer has been an important part of the NAEDI strategy, with access to ultrasound identified as one of four top priorities.^40^ Therefore, it was reassuring to find that participating GPs generally reported good access to diagnostic services. Concern was still expressed, however, about waiting times and the possibility that a primary care intervention may exacerbate pressure on secondary care services. One recent report^33^ showed that a sample of GPs (*n* = 402) perceived significant improvements in access to ultrasound, but one in four tests was still taking >1 month. Greater dissemination of information on diagnostic service capacity limitations will help GPs make an informed decision about participation in awareness-raising activities.

### Implications for research and practice

This survey shows that GPs are mainly supportive of projects to raise awareness of cancer but that they are concerned about the possible negative impact on patients, their own workload, and the availability of services in secondary care. There is a need to know more about the cost benefit of public cancer awareness interventions. An understanding of the impact on workload is vital if GPs are to commit time and money to such intervention.
